# CdSe QD Biosynthesis in Yeast Using Tryptone-Enriched Media and Their Conjugation with a Peptide Hecate for Bacterial Detection and Killing

**DOI:** 10.3390/nano9101463

**Published:** 2019-10-16

**Authors:** Vishma Pratap Sur, Marketa Kominkova, Zaneta Buchtova, Kristyna Dolezelikova, Ondrej Zitka, Amitava Moulick

**Affiliations:** 1 Department of Chemistry and Biochemistry, Mendel University in Brno, Zemedelska 1, CZ-61300 Brno, Czech Republic; vishmapratapsur@gmail.com (V.P.S.); kominkova.marketa@gmail.com (M.K.); ZanetaBurianova@email.cz (Z.B.); kriki.cihalova@seznam.cz (K.D.); zitkao@seznam.cz (O.Z.); 2 Central European Institute of Technology, Brno University of Technology, Purkyňova 656/123, 61200 Brno, Czech Republic

**Keywords:** biosynthesis, tryptone, QDs, cell-penetrating peptide, antibacterial

## Abstract

The physical and chemical synthesis methods of quantum dots (QDs) are generally unfavorable for biological applications. To overcome this limitation, the development of a novel “green” route to produce highly-fluorescent CdSe QDs constitutes a promising substitute approach. In the present work, CdSe QDs were biosynthesized in yeast *Saccharomyces cerevisiae* using a novel method, where we showed for the first time that the concentration of tryptone highly affects the synthesis process. The optimum concentration of tryptone was found to be 25 g/L for the highest yield. Different methods were used to optimize the QD extraction from yeast, and the best method was found to be by denaturation at 80 °C along with an ultrasound needle. Multiple physical characterizations including transmission electron microscopy (TEM), dynamic light scattering (DLS), energy-dispersive X-ray spectroscopy (EDX), and spectrophotometry confirmed the optical features size and shape distribution of the QDs. We showed that the novel conjugate of the CdSe QDs and a cell-penetrating peptide (hecate) can detect bacterial cells very efficiently under a fluorescent microscope. The conjugate also showed strong antibacterial activity against vancomycin-resistant *Staphylococcus aureus* (VRSA), methicillin-resistant *Staphylococcus aureus* (MRSA), and *Escherichia coli*, which may help us to cope with the problem of rising antibiotic resistance.

## 1. Introduction

Quantum dots (QDs) are nanoparticles with unique optoelectronic properties and exceptional resistance to photo and chemical degradation [1]. Currently, QDs are very important not only in solar cells and optoelectronic transistor components, but also in biological applications including biosensors, bio-imaging, and biomedical research. Cadmium-selenium (CdSe) QDs within a range of 1–20 nm are known to be used in in vivo imaging, diagnosis, and electro-optic devices [2,3,4]. The traditional synthesis processes of QDs generally need a high temperature and toxic reagents [5,6], which are contrary to the concept of green and sustainable chemistry. Presently, QDs biosynthesis has gained much attention because of the facile and natural reaction conditions, low cost, and efficient production with excellent properties [7]. The ability to biosynthesize nanoparticles has been reported previously in the case of several organisms like *Escherichia coli* [8,9,10], *Rhodobacter sphaeroides* [11], *Klebsiella pneumoniae* [12], *Klebsiella aerogenes* [13], *Gluconacetobacter xylinus* [14] yeast *Saccharomyces cerevisiae*, *Schizosaccharomyces pombe* [15], *Torulopsis* species [16], *Rhodosporidium diobovatum* [17], and in fungus, *Fusarium oxysporum* [18] and *Phanerochaete chrysosporium* [19]. The biosynthesis was also reported in psychrotolerant microorganisms [20], tumor cells [21], chloragogen cells of earthworms [10,22], and mouse and rat livers [23,24,25]; however, the methods need more improvisation for specific biological applications. The presence of microbial protein on the surface of the QDs can enhance their activities, which makes them attractive in medical research [26]. 

Bacterial infections have become a global public health issue with increased mortality, morbidity, and increased cost of treatment during patients’ hospital stay. In the last decade, the bacterial resistance towards commonly-used antimicrobial agents has become a major problem in veterinary and public health [27]. Bacterial resistance is rapidly increasing due to the misuse or extensive use of antibiotics, which urges the necessity to develop different novel antimicrobial agents [28]. The infections caused by the resistant bacteria are usually treated with antibiotics, which comes with side effects, with their incomplete dosage, and improper use can further assist in the development of the resistant against them, assisting it in being a global health problem. Thus, the importance of the development of new treatment strategies or alternatives to antibiotics against these multidrug-resistant bacterial strains comprises the major demands in 21st Century Medical science [29]

In the present work, CdSe QDs were biosynthesized in *Saccharomyces cerevisiae* cells using a novel method where we show for the first time that the concentration of tryptone highly affects the synthesis process. Different methods were used to optimize the extraction of the QDs from yeast cells including sonication, lysis at 90 °C, homogenization using mortar and pestle with liquid nitrogen, and denaturation at 80 °C + an ultrasound needle. Different physical characterizations including transmission electron microscopy (TEM), dynamic light scattering (DLS), energy-dispersive X-ray spectroscopy (EDX), and spectrophotometry were performed to study the optical features and size and shape distribution of the QDs. Finally, we develop a novel conjugate of the CdSe QDs along with a bacterial cell-penetrating peptide (hecate) for potential microbiological applications. The conjugate was tested for its ability to target bacterial cells and superior antimicrobial property against vancomycin-resistant *Staphylococcus aureus* (VRSA).

## 2. Experimental

### 2.1. Chemicals 

Cadmium chloride (CdCl_2_), sodium selenite (Na_2_SeO_3_), and other chemicals listed in the text were purchased from Sigma-Aldrich (St. Louis, MO, USA), meeting the specification of the American Chemical Society (ACS), unless stated otherwise. 

### 2.2. Biosynthesis of QDs in Saccharomyces Cerevisiae

*Saccharomyces cerevisiae* (ATCC 9763, Czech Collection of Microorganisms) cells were stored at −20 °C as a spore suspension in 20% (v/v) glycerol. Before use in this study, the strains were thawed and washed with distilled water to remove glycerol. The sterilization of the media was carried out for 30 min at 121 °C in a sterilizer (Tuttnauer 2450EL, Beit Shemesh, Israel). The prepared tryptone glucose yeast extract (TGY) (tryptone 20 g/L, yeast extract 10 g/L, and glucose 20 g/L) broth was inoculated with yeast into 25-mL Erlenmeyer flasks for 24 h on a shaker at 600 rpm and 30 °C. Different concentrations of tryptone were used in different samples of 22.5, 25, 27.5, and 30 g/L. Then, 4 mM Na_2_SeO_3_ was added to the yeast cells. Then, 1.3 mM CdCl_2_ was added at time intervals (6 h, 8 h, 12 h, 24 h) in separate replicas of the yeast cell cultures, and each replica was cultivated under dark with the same conditions for the next 20 h. After that, the medium with yeasts was centrifuged at 2000× *g* for 5 min, and fresh medium was added to the pellet and cultivated again for 15 h in light for a high amount of QDs and high fluorescence, while the other conditions were maintained in the same way. Each experiment was repeated 5 times. 

### 2.3. Absorbance and Fluorescence Spectrophotometry

The absorbance spectra of the samples were obtained from the spectrophotometer SPECORD 210 (Analytik Jena, Jena, Germany). The fluorescence properties of the samples were recorded using a microplate reader (Tecan Infinite M200 PRO, Grödig, Austria), according to the protocol from our previous paper [30]. Three hundred fifty- and four hundred fifty-nanometer excitation wavelengths were used for yeast cells and extracted QDs, respectively. 

### 2.4. Fluorescence Microscopy

The cells were imaged by using the inverted system microscope Olympus IX 71 (Olympus Corporation, Tokyo, Japan). The microscope was equipped with a mercury arc lamp, an X-cite 120 Lamp (120 W; Lumen Dynamics, Mississauga, Canada). The images were captured by the Camera Olympus DP73 and processed by Stream Basic 1.7 software. 

### 2.5. Studies on Antioxidant Activity and Glutathione Production

The antioxidant activity of yeasts was determined using DPPH (2,2-diphenyl-1-picrylhydrazyl), ABTS (2,2′-azino-bis(3-ethylbenzothiazoline-6-sulphonic acid), and an FRAP (ferric-reducing ability of plasma) method [31]. It is true that these three methods have been used for antioxidant activity measurement, but their approaches and principles are different. Hence, to get more accurate information about the free radicals’ formation, these three assays were performed and compared [32]. This is a standard approach for measuring antioxidant activity. The experiment was performed using a BS-400 automated spectrophotometer (Mindray, Shenzhen, China) following the manufacturer’s instruction. The determination of the reduced to oxidized glutathione ratio (GSH/GSSG) in yeast cells was performed using high-performance liquid chromatography with electrochemical detection (HPLC-ED), as previously described [33].

### 2.6. Extraction of the QDs from Yeast Cells

The release of QDs from the cell is required for their use. For further biological application and characterizations, the QDs needed to be extracted from the yeast cells. Yeast cells were ruptured by several separate or combined techniques like heat treatment, sonication, mortar-pestle crushing, etc. Firstly, the yeast cells were separated out from the media by centrifugation, washed thrice, and re-suspended in the same volume of water. After that, heat treatment was applied at 80 °C (in a dry heat block) [34] on yeast cells, and after heat shock, the cells were subjected to sonication at 4 × 5 min/2 min, pulse 80%, power 80% [35]. The cell lysis was also performed by liquid nitrogen and homogenization in a mortar and pestle following Dunn et al. [36], and we also tried to rupture the yeast cells by extreme heat treatment at 90 °C [34]. Then, different methods (sonication, lysis at 90 °C, homogenization using a mortar and pestle with liquid nitrogen, and denaturation at 80 °C + an ultrasound needle) were used to extract the QDs. The cell debris was separated from the supernatant using centrifugation (16,000× g for 10 min). Then, the supernatant was concentrated using an Amicon Ultra-15,100 K centrifugal filet apparatus (4000× g for 25 min). The QDs were further purified by dialysis against deionized ultrapure water [37,38]. 

### 2.7. Characterization of the Extracted QDs

Along with the fluorescence and absorbance study, dynamic light scattering (DLS) and transmission electron microscope (TEM) studies were also performed to characterize the extracted QDs (E:QDs). The average size of the E:QDs and size distribution were determined using quasielastic laser light scattering with a Malvern Zetasizer (NANO-ZS, Malvern Instruments Ltd., Worcestershire, U.K.) [39]. The TEM study was performed using a Tecnai F20 TEM (FEI, Eindhoven, Netherlands) [40]. Energy-dispersive X-ray spectroscopy (EDX) of the samples was performed according to the protocol of our previous work [41]. The photoluminescence quantum yield of the extracted QDs was calculated following the optical density at 450 nm (excitation wavelength) of Rhodamine 6G, and a similar value was set for QDs also. The main absorption peak of Rhodamine 6G and first absorption peak of QDs were kept under 0.1 to decrease the reabsorption of samples. The fluorescence quantum yields of quantum dots were calculated by comparing the integrated intensity of QDs and Rhodamine 6G [42]. The molar ration of Cd to Se was found to be 1:1.1.

### 2.8. Peptide Synthesis

The peptide hecate was synthesized using a Liberty Blue peptide synthesizer (CEM, Matthews, NC, USA). The synthesis, deprotection, and cleaving were carried out according to the protocol of our previous work [43,44]. Deblocking of the Fmoc protecting group was carried out with 20% piperidine v/v in N,N-dimethylformamide (DMF). The coupling process was performed using N,N,N′,N′-tetramethyl-O-(1Hbenzotriazol-1-yl)uronium hexafluorophosphate (HBTU), N,N-diisopropylethylamine (DIEA), and DMF. The cleavage of the side chain protecting groups was carried out by treatment with 95% trifluoroacetic acid (TFA) v/v, 2.5% H_2_O v/v, and 2.5% triisopropylsilane (TIPS) v/v under microwave irradiation for 30 min at 38 °C. The sequence of the peptide was FALALKALKKALKKLKKALKKAL [45].

### 2.9. Conjugation of the QDs with the Peptides 

The extracted QDs were diluted serially and incubated with our synthesized peptides in an Eppendorf tube. All the tubes were covered with aluminum foil and placed over night for rotation at room temperature on a rotator (Biosan Pvt. Ltd. Riga, Latvia), which was programmed at a 40-rpm speed with a 15-s interval of shaking. 

### 2.10. Application on Bacteria

VRSA (CCM 1767, Czech collection of microorganisms), MRSA (4750 Czech collection of microorganisms), and *E. coli* (ATCC 25922) were inoculated in Muller Hinton broth and placed in a shaking incubator overnight at 37 °C, for their substantial growth. From the overnight-grown culture, 200 µL of bacterial sample (0.1 Optical Density (O.D)) were taken in a 2-mL Eppendorf tube along with addition of 50 µL of each dilution of QDs + peptide conjugate and incubated for 24 h. The optical density of the applied agents was evaluated via the absorbance measurements using Multiskan EX (Thermo Fisher Scientific, Germany) according to our previous work [46]. Along with this experiment, we further performed the colony forming unit (CFU) experiment, where the positive control (untreated plate) had bacterial colonies and the negative control plate was formed with cefoxitin, which was without colonies [47]. The concentration of the cefoxitin was 32 µg/mL [48]. QDs conjugated with peptide mixed with Mueller Hinton (MH) broth and were inoculated with bacterial cultures adjusted to 1 × 10^8^. Then, the samples were incubated for 24 h in a shaker at 37 °C. After the incubation time period, 100 µL of bacterial samples treated with QD + peptide were serially diluted (10-fold) and plated on an MH agar plate and incubated at 37 °C for 24 h. The number of colonies was then multiplied by the dilution ration [44]. According to our previous work, we performed the broth micro dilution method for further confirmation and MIC determination [44].

### 2.11. Bacterial Cell Viability

The VRSA culture was treated with QDs + peptide conjugate for 24 h. Similar experiments with the control samples (bacteria without or with QDs or peptide) were also performed. The treatments were carried out in microplates. The total volume in the microplate wells was always maintained as 300 µL. All the experiments were repeated 5 times. Propidium iodide staining was performed according to our previous paper [44]. The number of cells observed in 10 randomized microscopic grid fields per sample was counted. The area of the ocular grid was 0.0156 mm^2^ [49,50]. 

### 2.12. Descriptive Statistics

Mathematical analysis of the data and their graphical interpretation were done by Microsoft Excel^®^, Microsoft Word^®^, and Microsoft PowerPoint^®^. Furthermore, the software Statistica (data analysis software system), Version 10.0 (StatSoft, Tulsa, OK, USA), was used for data processing. The general regression model was used to analyze the differences between the measured values. Unless noted otherwise, *p* < 0.05 was considered as significant [44]. Results are expressed as the mean ± the standard deviation (S.D.) unless noted otherwise. All the above-mentioned experiments were repeated 5 times.

## 3. Results and Discussion

### 3.1. Biosynthesis of CdSe QDs Using Tryptone-Supplemented Medium

**Figure 1:**
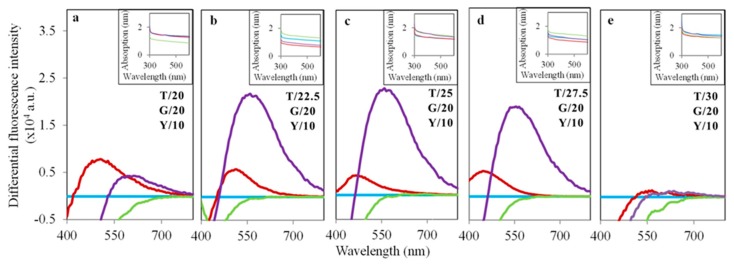
Differential fluorescence spectra (excitation at 350 nm) of yeasts cultivated in the Tryptone Glucose Yeast extract media (TGY) with the precursors for QD biosynthesis (CdCl_2_ and Na_2_SeO_3_). Different concentrations of tryptone (20–30 g/L) were added to the medium to optimize QD biosynthesis (**a**–**e**). The concentrations (g/L) of tryptone (T), glucose (G), and yeast extract (Y) are mentioned in each figure. Sky blue spectra denote the control (C) variant without the addition of CdCl_2_ and Na_2_SeO_3_. Red, green, and purple spectra represent the variant with the addition of only CdCl_2_ (Cd), Na_2_SeO_3_ (Se), and both (CdSe), respectively. Absorbance spectra are shown in the insets.

**Figure 2:**
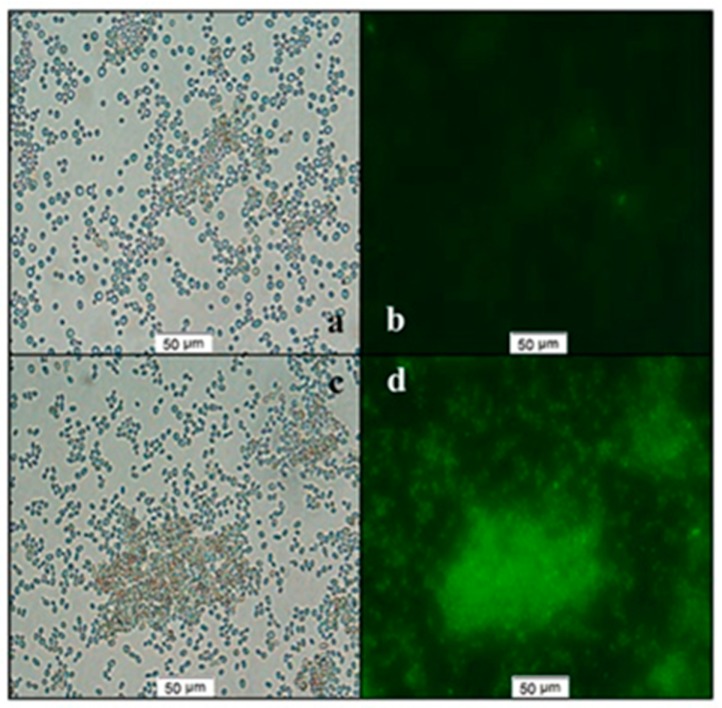
Bright-field and fluorescent micrograph of yeast cells with biosynthesized QDs. The yeasts were growing in normal TGY (**a**,**b**) and tryptone-supplemented TGY (**c**,**d**) medium with both precursors (CdCl_2_ and Na_2_SeO_3_).

The present experiment describes the biosynthesis of CdSe QDs in *Saccharomyces cerevisiae* using a tryptone-enriched medium. The yeast cells were initially grown in TGY medium for 24 h. After that, the medium was supplemented gradually with Na_2_SeO_3_ and CdCl_2_. The differential fluorescence spectra (excitation at 350 nm) of the yeasts cultivated only in TGY medium (C), medium with the addition of Na_2_SeO_3_ (Se), CdCl_2_ (Cd), and both Na_2_SeO_3_ and CdCl_2_ (CdSe) are shown in Figure 1. Without any addition of the precursors (CdCl_2_ and Na_2_SeO_3_), no quantum dot was found to be synthesized in the yeasts, whereas in the case of CdSe, a clear fluorescent spectrum was found with the emission maximum at 552 nm. The biosynthesis of the QDs was found to be significantly enhanced with the tryptone supplementation in the medium. To optimize the method, we added different amounts of tryptone (20, 22.5, 25, 27.5, and 30 g/L), yeast extract (10 g/L), and glucose (20 g/L). According to the results of fluorescent intensities (shown in Figure 2), 25 g/L of tryptone showed the highest production of the QDs in the yeast cells in the presence of both precursors (CdCl_2_ and Na_2_SeO_3_). Figure 2 represents the bright-field and fluorescent micrographs of the yeast cells without or with tryptone supplementation. 25 g/L of tryptone were used for all further experiments since this was found to be the optimized condition for highest QD production. According to the results, we found that the concentration of the tryptone had a significant effect on the synthesis of the CdSe QDs inside yeast cells, which suggests that tryptone plays an important role in the synthesis of the QDs. Tryptone has negatively charged amino acids, which possibly attracted the positively-charged Cd ions, and such a pseudo high concentration led to CdSe nucleation. High concentrations of tryptone may interact with the metabolic pathway and eventually reduce the QD production.

**Figure 3:**
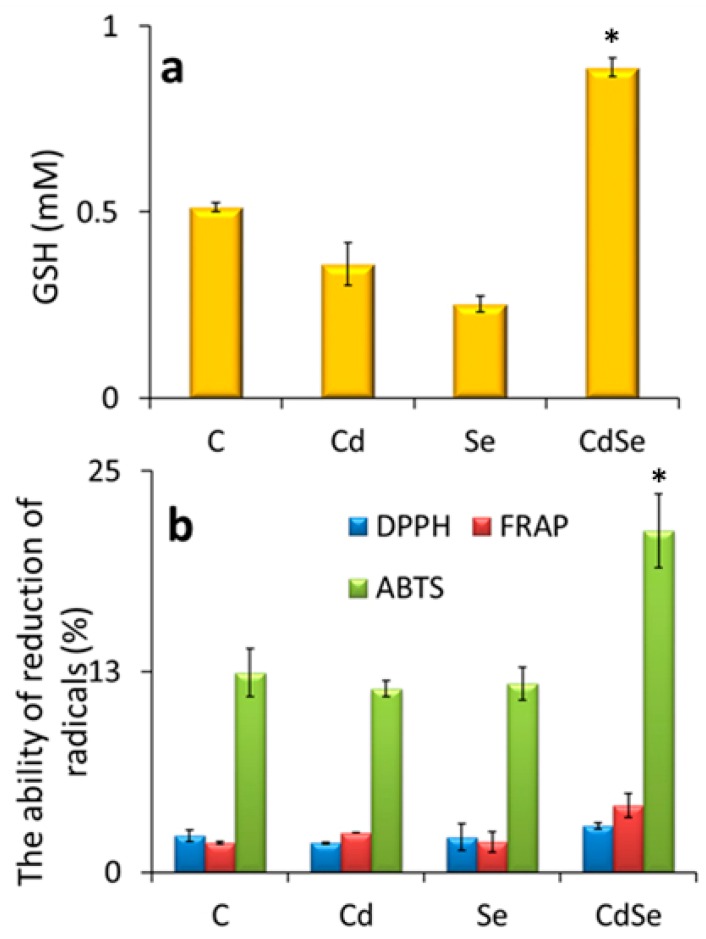
GSH production and antioxidant properties. (**a**) GSH production; (**b**) the antioxidant activity of the yeast cells (with or without the precursors) was determined during the QD biosynthesis. Data represent the mean ±SD, *n* = 5. Data represent the mean ±SD of the mean of three individual experiments. * *p* < 0.01 or ** *p* < 0.001.

It is important to identify the key biomolecules and metabolic pathways that are involved in the biosynthesis of CdSe QDs. Glutathione (GSH) is very important for CdSe QDs biosynthesis in yeast cells, which was previously proven by the deletion of some genes important in glutathione metabolism. As a ubiquitous tripeptide, glutathione (L-γ-glutamyl-cysteinyl-glycine) has a thiol group and a unique γ-glutamylcysteine peptide bond. These features facilitate the intracellular metabolism of inorganic compounds, which can be used to induce the in vitro synthesis of QDs. However, the intracellular correlation between the QD biosynthesis and glutathione metabolism is still not clear and somewhat controversial [51]. The GSH1 gene, which encodes γ-glutamylcysteine ligase (GCL), helps to catalyze the first and rate-limiting reaction of glutathione synthesis. To confirm its effect in QDs’ biosynthesis, the Δgsh1 mutant strain was produced by deleting the gene. The mutant showed a significant decrease of the production of QDs [51]. Similarly, the Δgsh2 mutant strain was also produced by deleting the GSH2 gene, which encodes glutathione synthetase, the catalyst of the second reaction of glutathione synthesis. Δgsh2 was not able to produce glutathione, though it could accumulate the dipeptide γ-glutamylcysteine, which has nearly all the features of glutathione. However, the mutant showed a significant decrease of QD biosynthesis. Deletion of both the genes (Δgsh1 Δgsh2 double mutant) also showed similar results, suggesting that the naturally-evolved structure and composition of glutathione are essential for CdSe QD biosynthesis, and they clearly mentioned that deletion of those genes were the cause of the fluorescence intensity decrease [51]. The oxidized form of glutathione (GSSG) is also present in yeast cells. The high ration reduced-to-oxidized glutathione is maintained by the gene GLR1. Δglr1 mutant cells were developed by deleting the gene to understand the role of the glutathione redox state in the biosynthesis of CdSe QDs. The gene deletion led to the accumulation of high levels of GSSG. In that case also, a significant reduction of the QD biosynthesis was observed, which led to the conclusion that the redox state of glutathione is an important factor for the biosynthesis [51]. In the present experiment, we determined the levels of reduced glutathione (Figure 3a), which is involved in metal homeostasis and their detoxification [33]. From all the variants, the highest level was observed in the case of the CdSe variant. Further, the antioxidant activity of the yeast cells (with or without the precursors) was determined during QD biosynthesis (Figure 3b). According to the results of the ABTS method, the yeast cells were showing the highest antioxidant activity during the incubation with both precursors.

Selenotrisulfide derivative of glutathione (GSSeSG), a reduced form of selenite, was produced by reduced glutathione (GSH) in yeast cells. GSSeSG is a substrate for GSH-related enzymes; for example, thioredoxin reductase and glutathione reductase can be reduced into low-valence organoselenium, selenomethionine, and selenocystine in yeast cells, which was already proven by HPLC-ICP-MS [52,53]. The selenite can further be reduced into selenium in the cytoplasm and mitochondria by GSH-associated enzymes, for example glutathione reductase and NADPH (reduced b-nicotinamide adenine dinucleotide 20-phosphate tetrasodium salt). When yeast cells are in the stationary phase, sodium selenite (Na_2_SeO_3_) is reduced into organoselenium complexes, such as selenocysteine and selenomethionine. GSH-related enzymes are considered as the key for selenium reduction [54,55]. On the other hand, Cd^+2^ can form bis(glutathionato)cadmium (CdSG2) complexes, which react with the reduced selenium to form CdSe QDs. Taken together, it can be concluded that the glutathione metabolic pathway is important for the biosynthesis of CdSe QDs [52]

### 3.2. Extraction of QDs from Yeast Cells and Their Characterization 

**Figure 4:**
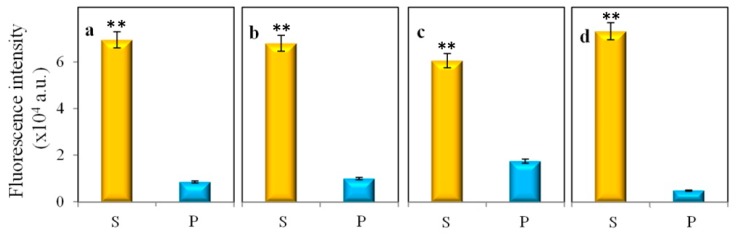
Comparison of fluorescence changes after cell lysis between different variants: (**a**) sonication; (**b**) lysis at 90 °C; (**c**) homogenization using a mortar and pestle and liquid nitrogen; and (**d**) denaturation at 80 °C + an ultrasound needle. The vertical axis represents the fluorescent intensity at the emission maximum at a wavelength of 536 nm. Four hundred fifty nanometers was used here as the excitation wavelength. The supernatant and pellet are designated as S and P, respectively. Data represent the mean ±SD, *n* = 5. Data represent the mean ±SD of the mean of three individual experiments. * *p* < 0.01 or ** *p* < 0.001.

**Figure 5:**
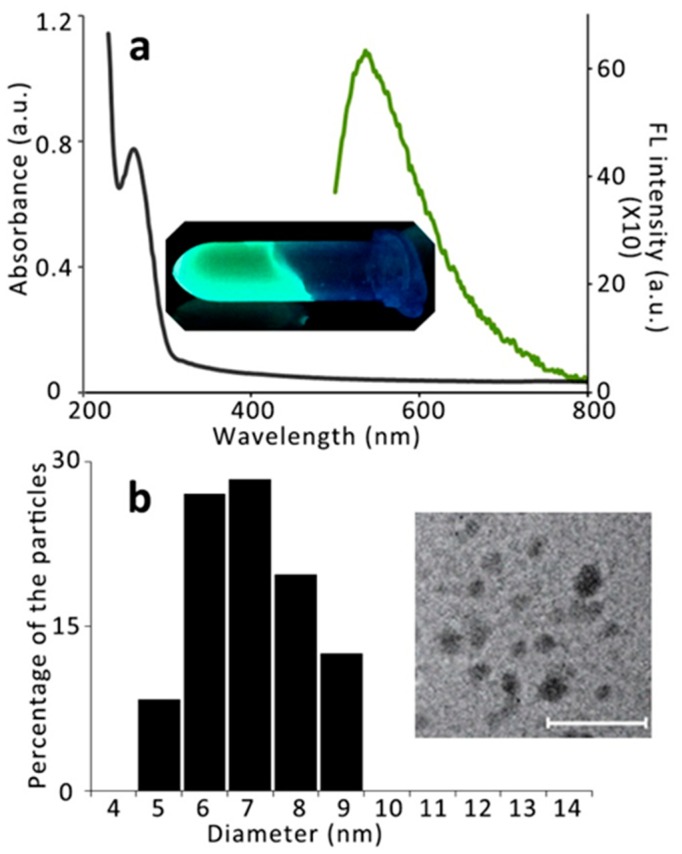
Characterization of extracted QDs (E:QDs). (**a**) Absorbance and fluorescence (FL) spectra are indicated by black and green colors, respectively. A photograph of the E:QDs under UV (λ = 312 nm) light is shown in the inset. (**b**) DLS measurements and size distribution. The column chart indicates the size of E:QDs. The TEM image of the E:QDs is shown in the inset. The scale bar is 40 nm.

The release of QDs from the cell is necessary for their use. For further biological application and characterizations, the QDs need to be extracted out from the yeast cells. Firstly, the yeast cells were separated out from the media by centrifugation, washed thrice, and resuspended in same volume of water. Then, different methods were used to extract the QDs (Figure 4). The cell debris was separated from the supernatant using centrifugation (16,000× *g* for 10 min). Then, the supernatant was concentrated using an Amicon Ultra-15,100 K centrifugal filter apparatus (4000× *g* for 25 min). The QDs were further purified by dialysis against deionized ultrapure water [37,38]. In each of the cases, we checked their fluorescent intensities at their emission maxima (536 nm), and 450 nm was used as the excitation wavelength. In all the cases, most of the synthesized QDs were found in the supernatant; however, the best yield was found in the case of denaturation at 80 °C + the ultrasound needle method. Therefore, this method was used for all further experiments. The absorbance and fluorescence spectra of the extracted QDs (E:QDs) are shown in Figure 5a. A photograph of the E:QDs under UV (λ = 312 nm) light is shown in the inset of Figure 5b. The extracted QDs were further characterized using dynamic light scattering (DLS) measurements and transmission electron microscopy (TEM) (Figure 5). The diameter of the extracted QDs was found to be 7 ± 2 nm according to DLS, which was in good agreement with the TEM results. The TEM image showed discrete CdSe QDs. The zeta potential of the sample was found to be −28 mV, which indicated that the stability of the QDs in the solution was good. EDX characterization of the E:QDs proved that the elements in the sample were Cd and Se (Figure S1, Supplementary Information). The highest fluorescence quantum yield for the E:QDs was 26.5% compared with control Rhodamine 6G.

### 3.3. Application of the Extracted QDs in Bacteria

**Figure 6:**
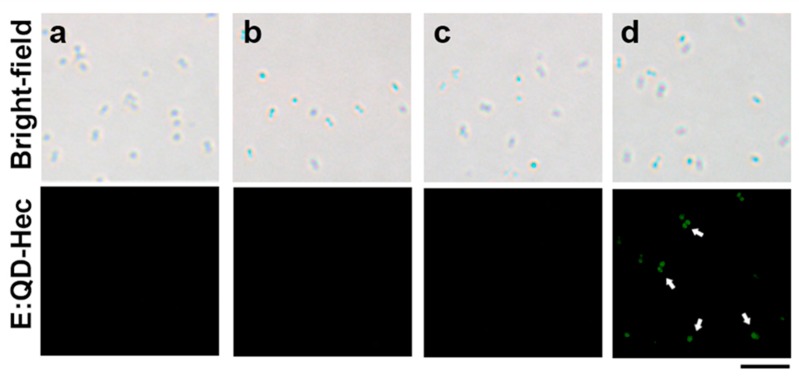
(**a**) Optical and fluorescence images of control VRSA where the bacteria were incubated in LB without any E:QD or Hec; (**b**) optical and fluorescence images of VRSA incubated only with Hec; (**c**) optical and fluorescence images of VRSA incubated only with E:QDs, with the fluorescence field; (**d**) optical and fluorescence images of VRSA incubated with E:QD-Hec conjugate with the fluorescence field. The scale bar is 5 µm.

In this experiment, we conjugated a synthetic peptide, hecate, with the E:QDs to study the detection and antibacterial activity. Hecate (Hec) was chosen since it is an antimicrobial peptide with a positive charge, which has the potential to interact with the negatively-charged surface of the bacteria. However, this peptide is unable to kill the vancomycin-resistant *Staphylococcus aureus* (VRSA) strain, which was published in our previous paper [44]. The E:QDs were added with the peptide solution (1mg/mL) in a ratio of 1:1 and mixed overnight at room temperature. The peptide is positively charged, and the QDs are negatively charged, so when they are incubated and rotated overnight, they bind with each other following Coulomb’s law. Unbound peptides were removed by dialysis. The E:QD-Hec conjugate was then applied on VRSA, MRSA, and *E. coli* and after 1 h of incubation at 37 °C, and the bacteria were washed thrice by centrifugation using Luria-Bertani (LB) broth media to remove the unbound E:QD-Hec. Then, the bacteria were observed under a fluorescent microscope. Similar experiments with the control samples (bacteria without or with E:QD or Hec) were also performed. According to the result of fluorescent microscopy (Figure 6), the bacteria with the E:QD-Hec conjugate showed bright green fluorescent, whereas none of the control bacteria showed any fluorescent, which was probably due to the cell-penetrating property of the peptide [44]. This result clearly proved that the E:QD-Hec conjugate can detect the bacteria successfully. 

**Figure 7:**
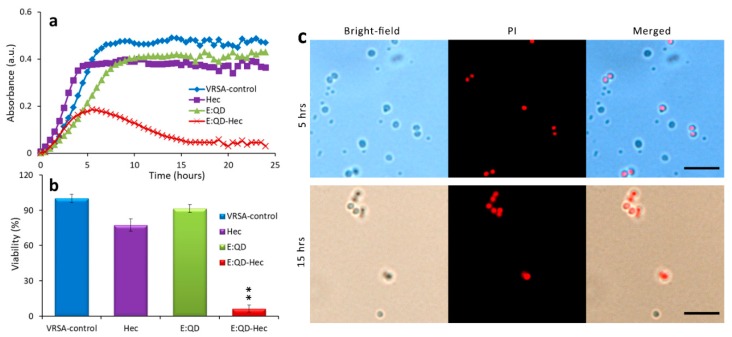
Antibacterial property of the QD-peptide conjugate. (**a**) Growth curve analysis of VRSA. The growth of the bacteria is represented in terms of absorbance (a.u.). (**b**) Bacterial cell viability after 24 h of incubation. The viabilities of the samples are represented here as the percentage of VRSA-control (only the bacteria that were incubated without Hec or E:QDs). Hec, E:QD, and E:QD-Hec denote the bacterial samples incubated with only the peptide, only E:QDs, and E:QD-peptide conjugate, respectively. Data represent the mean ± SD, *n* = 5. (**c**) Bright-field and fluorescent microscopic images of VRSA after 5 and 15 h of treatment with E:QD-Hec. The dead cells are seen as red fluorescence from propidium iodide (PI). The scale bar is 5 µM. Data represent the mean ± SD of the mean of three individual experiments. * *p* < 0.01 or ** *p* < 0.001.

Further, to check the antibacterial properties, we incubated VRSA, MRSA, and *E. coli* with the E:QD-Hec conjugate as before, but the incubation time was set at 24 h in this case. The bacterial growth was monitored using the absorbance every 30 min during the total incubation period. Control experiments were performed simultaneously. According to the results (Figure 7a, Figure S2), it was clearly seen that only E:QDs were unable to show substantial inhibition of VRSA growth, which is in good agreement with some previously-published results. Only Hec was also unable to show a considerable change in bacterial growth. These data are in good agreement with previously-published results [44,56]. However, the E:QD-Hec conjugate was able to show significant inhibition of bacterial growth, which started gradually after 6 h of incubation. After 24 h of incubation with E:QD-Hec (at different concentration dilutions), the cell viability was found to be only 6.39% as compared to the control (Figure 7b). Furthermore, we performed a dose-dependency test where we applied different dilution concentrations of QDs conjugated with hecate applied on bacterial samples and monitored by thermo Multiskan for the next 24 h, and results clearly indicated that our QDs conjugated with peptide had very good antibacterial properties (Figures S2–S7). Bright-field and fluorescent microscopic images of VRSA after 5 and 15 h of treatment with E:QD-Hec are shown in Figure 7c. The dead cells are seen as red fluorescence from propidium iodide (PI). It is clearly seen from the Figure 7a that the bacterial growth was significantly inhibited by the 15 h of treatment. All these data suggested that the E:QD-Hec conjugate was a powerful antibacterial agent against the antibiotic-resistant bacterial strain. According to our knowledge, only CdSe did not show any effects against VRSA or MRSA. The peptide hecate itself was unable to show any significant antibacterial effect against VRSA or MRSA. However, it showed a significant effect against antibiotic-resistant bacterial strains after conjugation with some compounds (e.g., vancomycin), which may cause its structural changes and help it to interact with the bacterial cell wall [44]. Hecate at a lower concentration has been found to be nontoxic to human cells [44]. We confirmed bacterial elimination with E:QD-Hec by a CFU experiment where bacterial viability for the control plate of VRSA was 4.1 × 10^7^ CFU/mL and for the treated plate was 0.09 × 10^7^, MRSA 3.9 × 10^7^. However, for the treated plate, no colonies were observed, while in the case of the *E. coli* control plate, the CFU value was 5.6 × 10^7^, and the treated cell plate contained no bacterial colonies (Figure S8 (VRSA), Figure S9 (*E.coli*); MRSA showed the same pattern as VRSA). MIC was determined (broth microdilution) for all three bacteria used with a half fold dilution of QD conjugated with peptide. However, quantum dots are now used in in vivo applications such imaging [57,58] and drug delivery in the future. They can be used for diagnostic purposes as well. However, it is still unclear how peptide conjugated ones can be used. In our previous work, we showed that only hecate (Hec) was not effective against resistant strains of *Staphylococcus aureus* like VRSA, but it showed very good activity when conjugated with antibiotic, which was not effective against VRSA. On the other hand, peptide conformation plays an important role in interacting with bacteria [59,60]. After the conjugation with the E:QDs, the conformation of the peptide was probably changed in such a way that helped it to interact with the bacteria. The peptide hecate itself was unable to show any significant antibacterial effect against VRSA or MRSA. However, it showed a significant effect against antibiotic-resistant bacterial strains after conjugation with some compound (e.g., vancomycin), which may cause its structural changes and help it to interact with the bacterial cell wall [44] 

## 4. Conclusions

In the presented work, CdSe QDs were successfully biosynthesized in living *Saccharomyces cerevisiae* cells using a novel method, where tryptone glucose yeast extract medium (TGY) was used along with the precursors (CdCl_2_ and Na_2_SeO_3_). The obtained QDs were successfully extracted from the cells with a high yield and subsequently characterized. The size of the green fluorescent QDs was found to be 7 ± 2 nm (diameter). The highest fluorescence quantum yield for the extracted QDs was 26.5%. This is the first report to show that tryptone supplementation in TGY medium can significantly induce the biosynthesis of QDs. On the basis of microscopic images, the bacterial growth curve, CFU, and broth microdilution, it can be concluded that the QDs, after their conjugation with a cell-penetrating peptide, can detect bacterial cells and show significant antibacterial activity against vancomycin-resistant *Staphylococcus aureus* (VRSA), methicillin-resistant *Staphylococcus aureus* (MRSA), and *Escherichia coli* (*E. coli*), and with propidium iodide, we quantified dead cells according to the number of cells observed in 10 randomized microscopic ocular grid fields per sample. The area of the ocular grid was 0.0156 mm^2^.

## Supplementary Materials

The following are available online at https://www.mdpi.com/2079-4991/9/10/1463/s1: Figure S1: EDX, Figure S2: Growth curve VRSA, Figure S3: Growth Curve MRSA, Figure S4: Growth Curve E. Coli, Figure S5: VRSA viability count, Figure S6: MRSA viability count, Figure S7: E. coli viability count, Figure S8: CFU VRSA, Figure S9: CFU E. coli.
