# Supplementary material for: CdSe QD Biosynthesis in Yeast Using Tryptone-Enriched Media and Their Conjugation with a Peptide Hecate for Bacterial Detection and Killing
Source: Nanomaterials (Basel). 2019 Oct 16;9(10):1463. doi: 10.3390/nano9101463 (PMC6835635; doi:10.3390/nano9101463)
Supplement: Supplementary file 1 [file nanomaterials-09-01463-s001.pdf]

Supplementary Information for

CdSe QD Biosynthesis in Yeast Using Tryptone-Enriched Media and Their Conjugation with a Peptide Hecate for Bacterial Detection and Killing

Vishma Pratap Sur [1,2], Marketa Kominkova [1], Zaneta Buchtova [1,2], Kristyna Dolezelikova [1], Ondrej Zitka [1,2] and Amitava Moulick [1,2,*]

1 Department of Chemistry and Biochemistry, Mendel University in Brno, Zemedelska 1, CZ-61300 Brno, Czech Republic; vishmapratapsur@gmail.com (V.P.S.); kominkova.marketa@gmail.com (M.K.); ZanetaBurianova@email.cz (Z.B.); kriki.cihalova@seznam.cz (K.D.); zitkao@seznam.cz (O.Z.)

2 Central European Institute of Technology, Brno University of Technology, Purkyňova 656/123, 61200 Brno, Czech Republic

* Correspondence: amitava.moulick@mendelu.cz, amitavamoulick@gmail.com; Tel.: +420-54513-3350

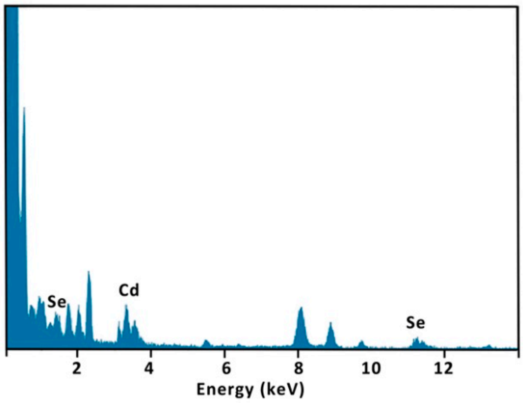

Figure S1: Energy –dispersive X-ray spectroscopy (EDX) spectrum of the extracted CdSe QDs

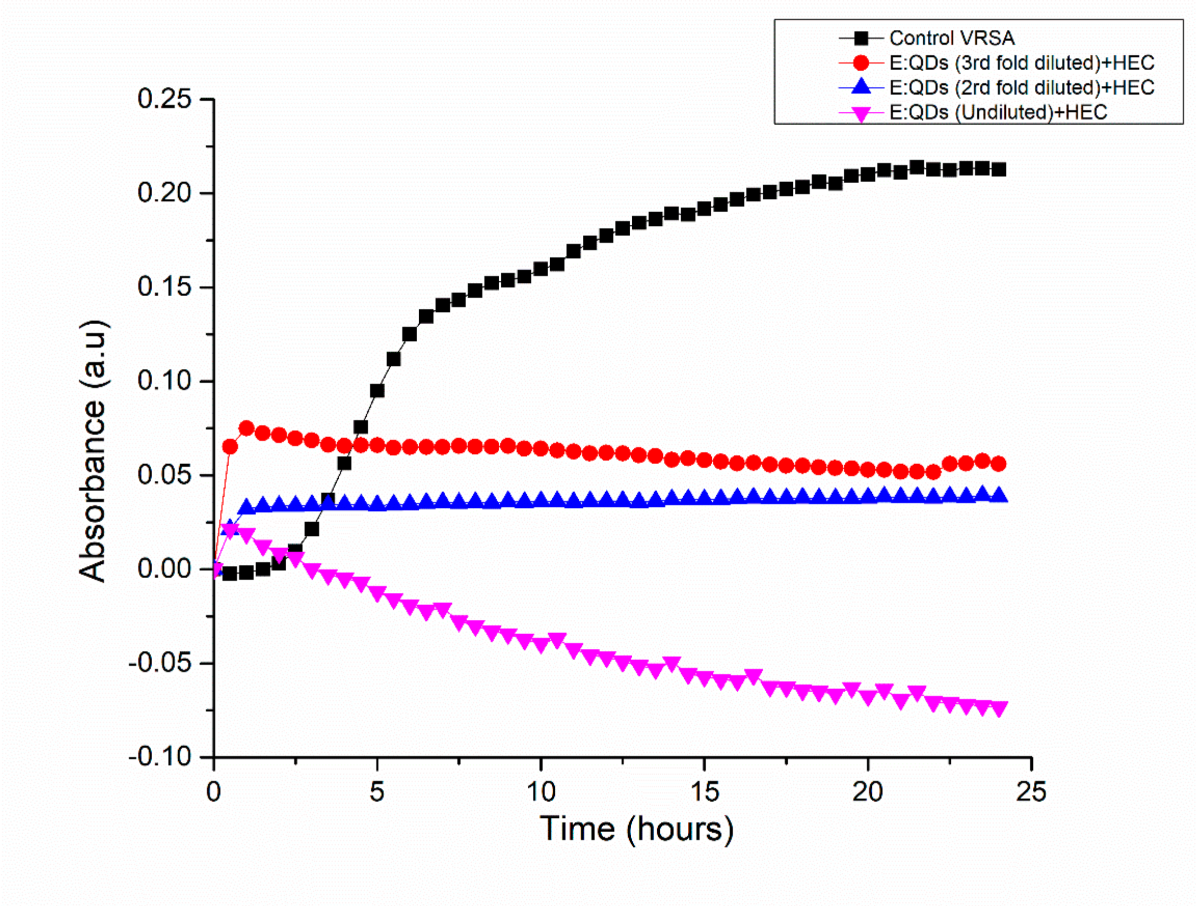

Figure S2: Bacterial growth curve analysis. VRSA Growth curve against E:QDs (3rd fold diluted) + HEC, E:QDs (2rd fold diluted) + HEC, E:QDs (Undiluted) + HEC. Data represent the mean ±SD, *n* = 5.

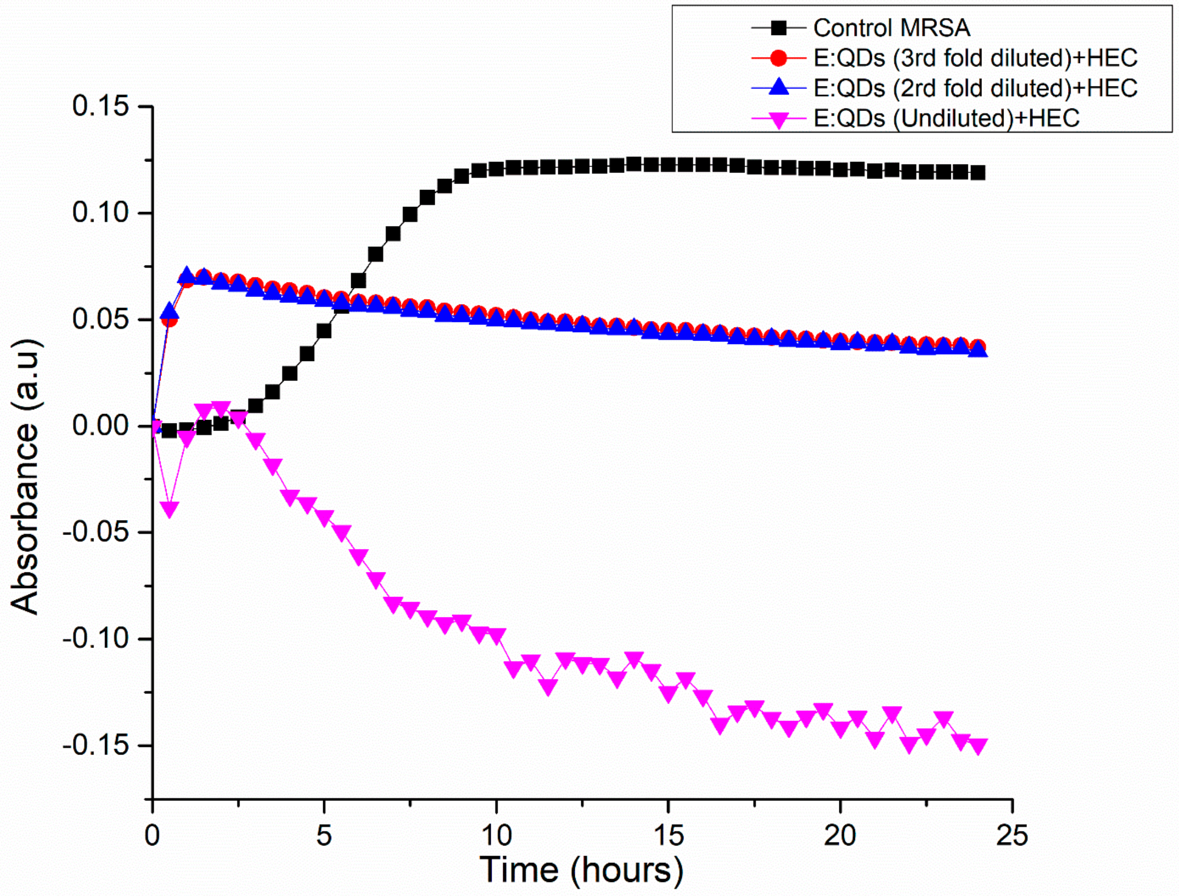

Figure S3: Bacterial growth curve analysis. MRSA Growth curve against E:QDs (3rd fold diluted) + HEC, E:QDs (2rd fold diluted) + HEC, E:QDs (Undiluted) + HEC. Data represent the mean ±SD, $n = 5$.

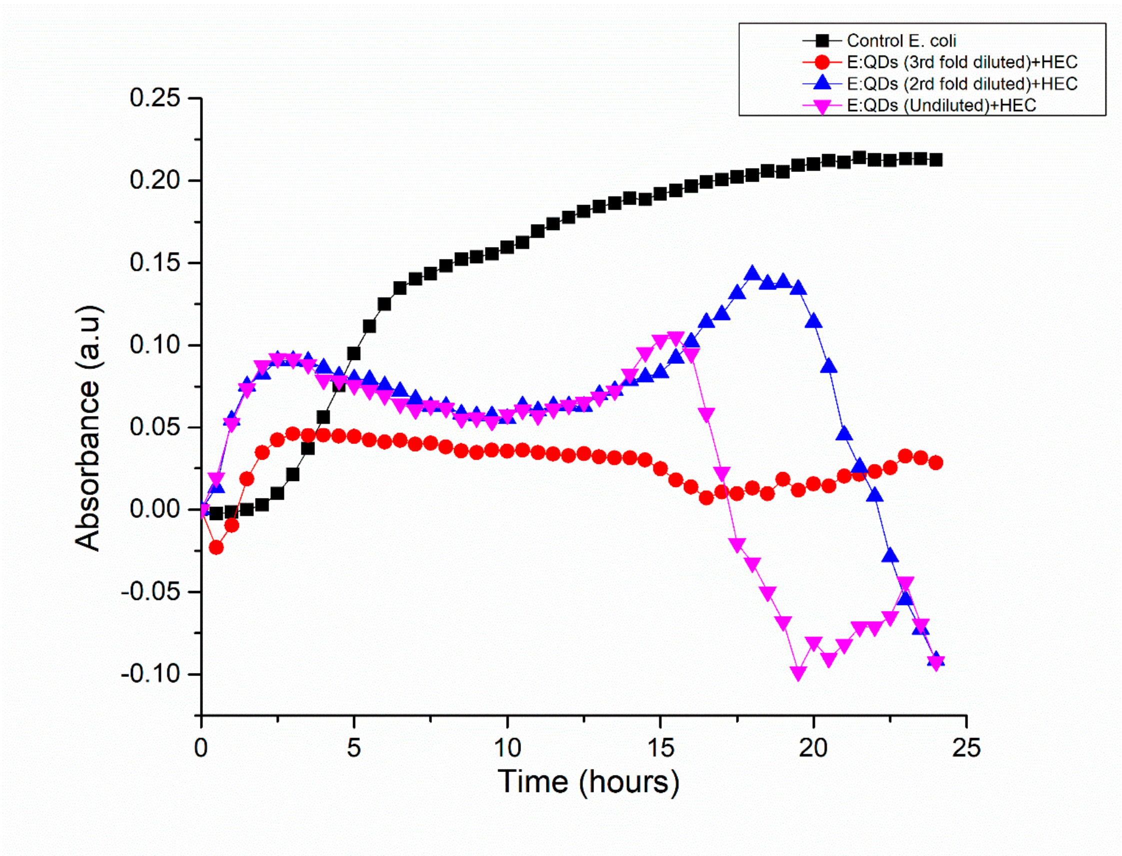

Figure S4: Bacterial growth curve analysis. E. coli Growth curve against E:QDs (3rd fold diluted) + HEC, E:QDs (2rd fold diluted) + HEC, E:QDs (Undiluted) + HEC. Data represent the mean ±SD, n = 5.

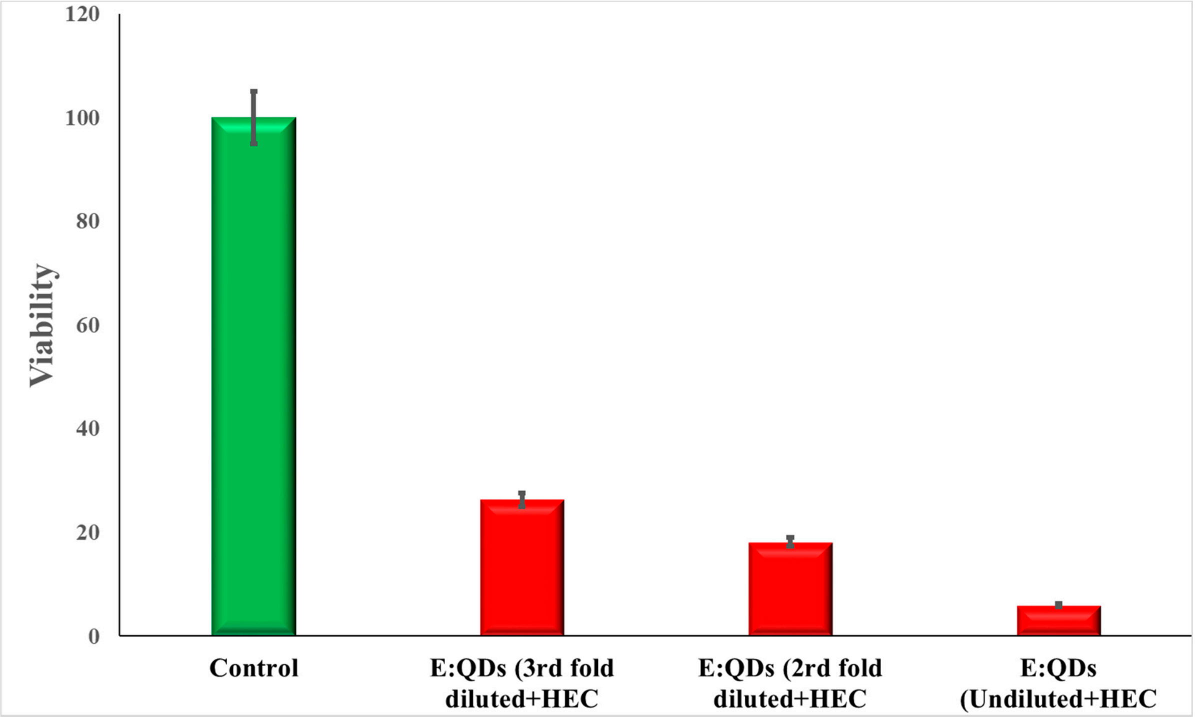

Figure S5: Viability count of VRSA: The representative viability count of VRSA against E:QDs (3rd fold diluted) + HEC, E:QDs (2rd fold diluted) + HEC, E:QDs (Undiluted)+ HEC. Data represent the mean ±SD, $n = 5$.

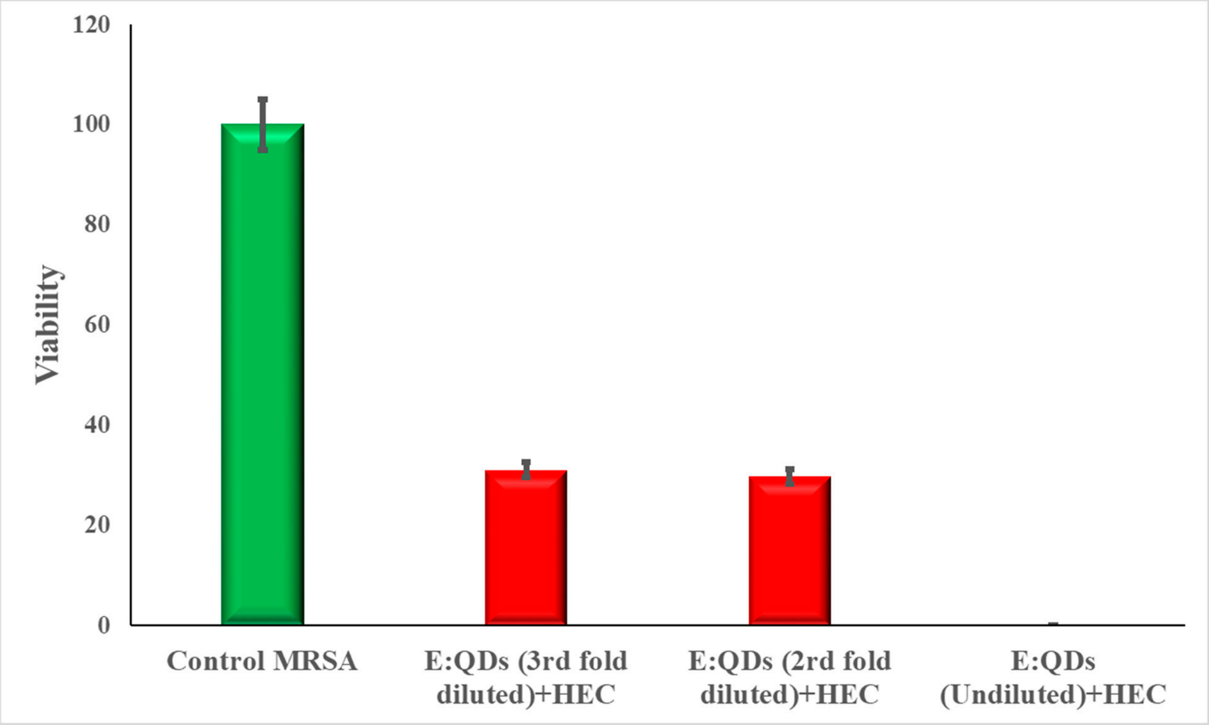

Figure S6: Viability count of MRSA: The representative viability count of MRSA against E:QDs (3rd fold diluted) + HEC, E:QDs (2rd fold diluted) + HEC, E:QDs (Undiluted) + HEC. Data represent the mean ± SD, *n* = 5.

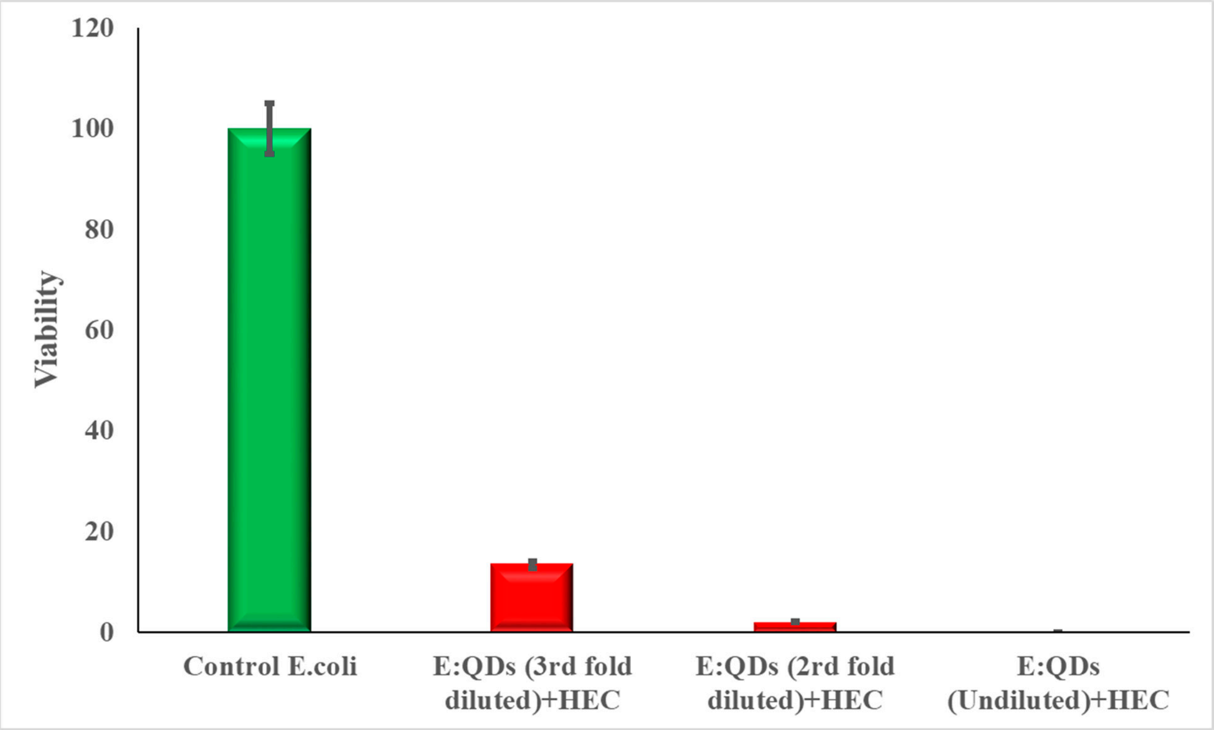

Figure S7: Viability count of E. coli: The representative viability count of E. coli against E:QDs (3rd fold diluted) + HEC, E:QDs (2rd fold diluted) + HEC, E:QDs (Undiluted) + HEC. Data represent the mean ± SD, $n = 5$.

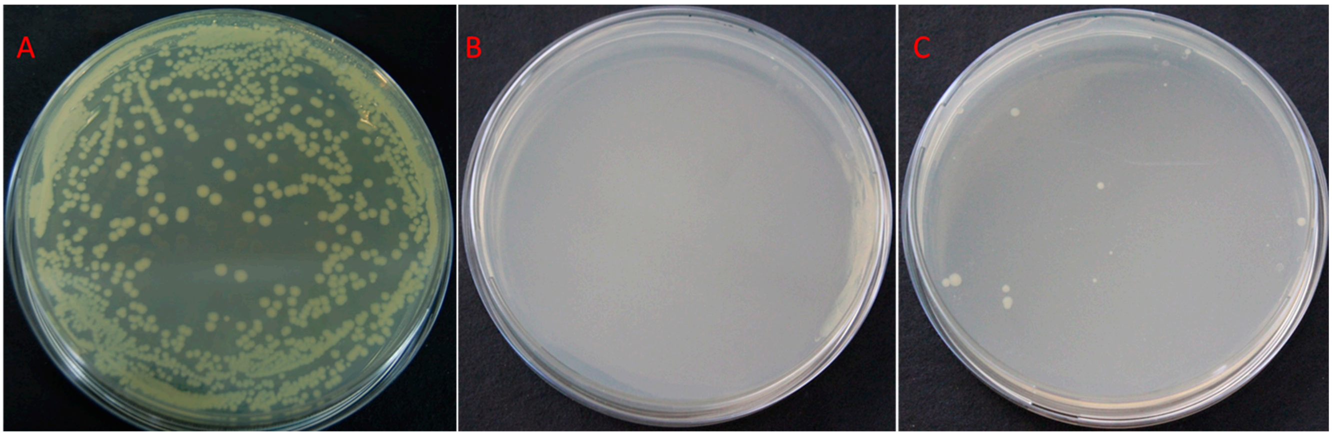

Figure S8: Colony forming units of VRSA. Where (A) positive control plate, (B) negative control plate and (C) treated with E:QD-peptide conjugate.

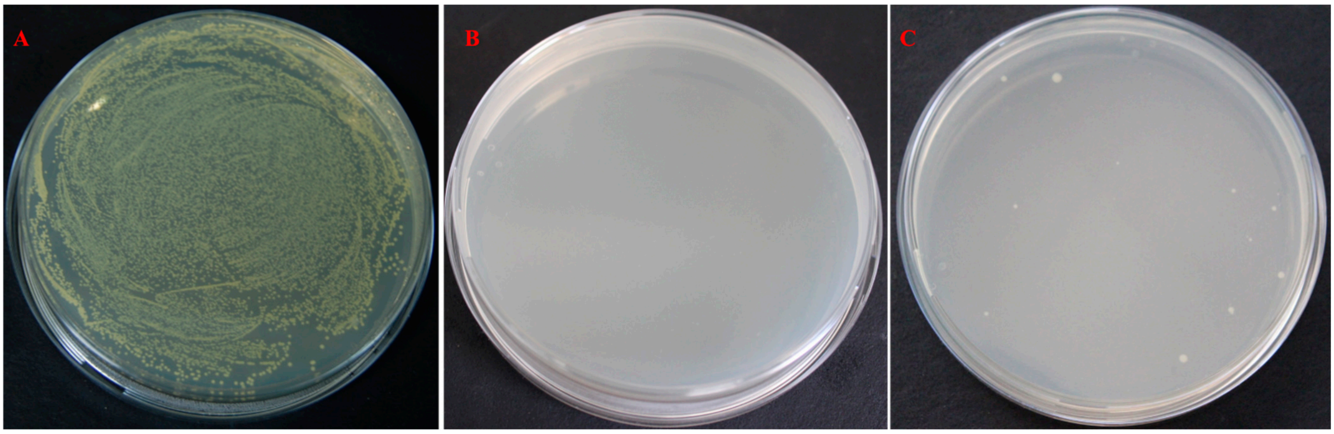

Figure S9: Colony forming units of E. coli. Where (A) positive control plate, (B) negative control plate and (C) treated with E:QD-peptide conjugate.
